# ‘Everything was just getting worse and worse’: deteriorating job quality as a driver of doctor emigration from Ireland

**DOI:** 10.1186/s12960-019-0424-y

**Published:** 2019-12-09

**Authors:** N. Humphries, A. M. McDermott, E. Conway, J-P Byrne, L. Prihodova, R. Costello, A. Matthews

**Affiliations:** 1 Research Royal College of Physicians of Ireland, Dublin, Ireland; 2 Cardiff Business School, Cardiff University, Cardiff, UK; 3 Dublin City University Business School, Dublin City University, Dublin, Ireland; 4 Royal College of Surgeons in Ireland, Dublin, Ireland; 5 School of Nursing, Psychotherapy and Community Health, Dublin City University, Dublin, Ireland

**Keywords:** Medicine, Migration, Job quality, Austerity, Qualitative, Extreme work

## Abstract

**Background:**

Medicine is a high-status, high-skill occupation which has traditionally provided access to good quality jobs and relatively high salaries. In Ireland, historic underfunding combined with austerity-related cutbacks has negatively impacted job quality to the extent that hospital medical jobs have begun to resemble extreme jobs. Extreme jobs combine components of a good quality job—high pay, high job control, challenging demands, with those of a low-quality job—long working hours, heavy workloads. Deteriorating job quality and the normalisation of extreme working is driving doctor emigration from Ireland and deterring return.

**Methods:**

Semi-structured qualitative interviews were conducted with 40 Irish emigrant doctors in Australia who had emigrated from Ireland since 2008. Interviews were held in July–August 2018.

**Results:**

Respondents reflected on their experiences of working in the Irish health system, describing hospital workplaces that were understaffed, overstretched and within which extreme working had become normalised, particularly in relation to long working hours, fast working pace, doing more with less and fighting a climate of negativity. Drawing on Hirschman’s work on exit, voice and loyalty (1970), the authors consider doctor emigration as exit and present respondent experiences of voice prior to emigration. Only 14/40 respondent emigrant doctors intend to return to work in Ireland.

**Discussion:**

The deterioration in medical job quality and the normalisation of extreme working is a key driver of doctor emigration from Ireland, and deterring return. Irish trained hospital doctors emigrate to access good quality jobs in Australia and are increasingly likely to remain abroad once they have secured them. To improve doctor retention, health systems and employers must mitigate a gainst the emergence of extreme work in healthcare. Employee voice (about working conditions, about patient safety, etc.) should be encouraged and used to inform health system improvement and to mitigate exit.

## Background

Research into doctor migration indicates that it is driven by a combination of push factors which encourage doctors to emigrate from their country of origin (or country of training), and pull factors, which attract them to specific destination countries. Early research into doctor migration found that it tended to be driven by poor medical career advancement opportunities at home, a desire for specialist training opportunities abroad [1] and the desire to enhance one’s professional and financial situation [2].

Doctor emigration poses a challenge to all health systems in that it has an immediate impact on the supply of doctors into the workforce [3], but it has a particularly negative impact on weak or fragile health systems [4, 5]. The 2010 Global Code developed by the World Health Organization (WHO) sought to mitigate the negative impact of health worker migration on fragile health systems and developing countries [6] by encouraging member states to strive, wherever possible, ‘to meet their health personnel needs with their own human resources for health’ [6].

While the drivers of migration have remained remarkably consistent over time (i.e. doctors still move to access better wages, better working conditions and better career prospects), the direction of migration flows change in response to local and global economic conditions. For instance, the 2008 global economic recession and resultant austerity measures—including widespread pay cuts, recruitment embargoes, staff reductions and increased workloads [7]—led to high rates of emigration and intent to migrate among doctors in higher income countries across the European Union (EU), including Ireland [8–10], Hungary [11], Romania [12] and Portugal [13].

Although medicine is a high-status, high-skill occupation which has traditionally provided access to good quality jobs and relatively high salaries, continuing to provide such benefits in the context of an economic recession and period of austerity is a challenge. Health systems and health employers must adapt to today’s expectations of what constitutes a good quality medical job [14], and do so within funding constraints, either austerity-related and/or, as in Ireland, those resulting from ‘historic long-term underfunding’ [15].

Drawing on interviews with Irish hospital doctors in Australia, this paper considers how deteriorating job quality and the normalisation of extreme working is driving Irish trained hospital doctors to exit [16] the Irish health system via emigration [9] and is also deterring their return to work in the Irish health system [8]. In the context of Brexit-driven uncertainty [17], and a global shortage of health professionals [6], the paper offers timely international insights for health service employers and policy makers, regarding the importance of addressing deteriorating job quality in order to retain hospital doctors.

### Response to deteriorating job quality

Job quality is ‘the extent to which a job has work and employment-related factors that foster beneficial outcomes for the employee, particularly psychological wellbeing, physical wellbeing and positive attitudes’ [18]. Good-quality jobs also benefit employers, increasing average tenure and productivity [19]. Work organisation (including demands and resources), wages and payment systems, security and flexibility (e.g. contracts, working hours and flexibility), skills and development (e.g. skill use, training), and engagement (e.g. consultation and voice) have been identified as five main dimensions affecting job quality [18]. Jobs are typically classified as being of good, moderate or poor quality, although job characteristics may vary across employers, and the same job may be good in some respects but not others [19].

‘Extreme jobs’ combine components of a good quality job—high pay, high job control, challenging demands, with those of a low-quality job—long working hours and very high workloads [18]. In industry, individuals are considered to hold extreme jobs if they work more than 60 h per week, are high earners and have jobs meeting five of ten extreme work characteristics [20]. A healthcare variant of an extreme job, developed via research with hospital managers [21], is characterised by long hours and physical presence; tight deadlines and a fast working pace; unpredictable workflow; inordinate scope of responsibility; 24/7 availability; and responsibility for mentoring staff. Further healthcare-specific dimensions of an extreme job identified [22] include making life or death decisions, managing conflicting and changing priorities, being required to do more with fewer resources, responding to regulatory bodies and fighting a climate of negativity. Those who hold extreme jobs may be motivated and challenged by this way of working [22], enjoying the high pay, recognition, status, power [20] and exhilaration [19] that accompany extreme work. However, extreme jobs can also have a negative impact on employee wellbeing and on family relationships [20], with half of those in extreme roles indicating that they do not want to keep working under such pressure [20]. This has led to an acknowledgement of the risks associated with extreme work [20] and suggestions that extreme jobs—or extreme work—are unsustainable in the longer term [21].

The content of jobs and expectations of what constitutes a good-quality job can change over time and in response to wider social and economic conditions. For instance, austerity measures have shaped work by decreasing health budgets, reducing staffing levels and causing workloads to increase. This has contributed to the ‘growing sense in which “normal” workplaces are becoming “extreme” ’[21], particularly in the public sector post-2008 [23]. Wider cultural change can also alter perceptions of job quality. For instance, a professional culture ‘that celebrates the extreme ethos today may tire of it . . . tomorrow’ [20]. Hewlett and Luce warn that a failure to adapt to changing work preferences can pose a significant risk, including the risk that workers stop striving for top jobs which are no longer considered attractive [20]. Perceptions of job quality are also influenced by what is available in other countries. For health professionals whose skills are in high demand globally, the job quality (and salary levels [3]) on offer internationally also inform emigration decisions [8, 24, 25]. For instance, Australia escaped the worst effects of the global economic crisis of 2008, making it an attractive destination [9].

Workers face choices when dissatisfied with their job quality. Here we turn to Hirschman and his theory of exit, voice and loyalty [16]. Hirschman developed this theory to explain consumer choices when faced with a decline in service. His later iterations of the theory applied it to the relationship between citizens and the state and considered how states and organisations might seek to manipulate exit or voice [26, 27]. Following Hirschman [16], we note that when dissatisfied with their working conditions in the Irish health system doctors can opt for loyalty, i.e. remain working in the health system, they can try to improve things by speaking up (voice), or they can emigrate (exit) [16]. Or, as discussed by Hirschman, they can attempt a combination of these options. In this paper, we focus on emigrants, who by definition have opted for exit, although we also take account of voice, in recognition of the fact that exit and voice can operate independently, sequentially or can co-occur (e.g. via ‘noisy’ exit) [28]. Workers are more likely to use voice when the costs are low, when they can remember being happy in their job, have organisational commitment and believe that improvement is possible [28]. Workers are more likely to exit when they are dissatisfied, the costs of exit are low, the costs of voice are high, improvement is considered unlikely and the alternatives are attractive [28]. Crucially, for high-skill, in-demand workers, such as doctors, emigration offers a clear exit from system-wide deterioration in job quality. We consider medical migration as exit and discuss the risks that this poses to the Irish health system.

### Doctor migration

Medicine offers doctors a ‘boundaryless global career’ [29], an attractive prospect for the individual doctor, but one which can present challenges at a national level. Indeed, concern with ‘brain drain’ has extended from low- to middle-income countries (LMIC) [30], to high-income countries as austerity has impacted health budgets, including in Ireland, Germany, Portugal, Spain, Greece and the UK [31, 32]. As a result, some of these countries have become increasingly concerned about doctor emigration. The fear, recently expressed by the German Minister for Health [33], is that national workforce policies ‘may be undermined by the pay levels, job opportunities and workforce policies in other countries’ [34]. NHS England is currently short of more than 100,000 staff, with workforce challenges—including doctor shortages—considered a greater threat to care delivery than funding challenges [35]. In the UK, recent autobiographical literature has focussed attention on difficult working conditions for hospital doctors in the NHS [36, 37] citing them as drivers of doctor emigration such that ‘every rota bears the scars of doctors who’ve activated their Plan B—working in Canada or Australia’ [36]. Thus, the 2008 global economic crisis changed the context for migration [38], altering the direction and strength of health worker migration flows [34], both within the European Union (EU) and globally.

### Doctor emigration from Ireland

Irish doctors have a long tradition of emigration. The established pattern was to emigrate for 1–2 years to complete a fellowship in the USA or the UK, before returning to work in Ireland. As doctors returned in sufficient numbers to ensure strong competition for consultant posts, emigration was widely considered beneficial [39], rather than potentially harmful, to the Irish health system. A strong culture of medical migration resulted and emigration was promoted ‘as an essential (rather than an optional) component of a successful medical career’ [40].

The dynamics of doctor emigration changed, post-2008, as the Irish health system endured ‘radical resource cuts’ [32], including a 13% reduction in both staffing levels and acute hospital beds [41] and significant pay reductions. Salaries for new entrant hospital consultants were reduced by 10% in 2011 and by a further 30% in 2012 [42]. A 2018 court settlement further widened the salary differential between existing and new entrant hospital consultants [42]. Reduced salaries and deteriorating working conditions since 2008 have driven a high rate of doctor emigration from Ireland. They have also deterred return migration, with one in four emigrant doctors surveyed in 2014 intent on return [8]. Although Ireland does not formally record emigrant departures [43], researchers have estimated that at least 627 doctors emigrated from Ireland in 2014 [40] (in a year when 684 Irish/EU doctors graduated from Irish medical schools [44]). Recent data from the Medical Council of Ireland shows a year on year increase in doctors exiting the medical register—from 546 in 2014 to 1453 in 2018 [45]. Australia has emerged as a key destination for Irish trained doctors and issued 326 visas to Irish doctors in 2017–2018 [9].

Drawing on 40 interviews with emigrant Irish doctors who moved to Australia since 2008, this paper considers how deteriorating job quality and the normalisation of extreme working drive emigration—conceptualised as exit. Possibilities for voice [16, 26, 27] are also discussed. Only 14/40 respondents intend to return to work in the Irish health system, indicating, firstly, the extent to which the traditional pattern of return migration has changed and, secondly, the extent to which doctor emigration (without return) poses a significant risk to the Irish health system.

## Methods

This paper is the first in a series of papers^1^ from the first work-package of the HRB-funded Hospital Doctor Retention and Motivation Project (EIA-2017-022) which draws on qualitative interviews with Irish trained doctors in Australia (*N* = 51). This paper focusses on a subset of doctors (*N* = 40), i.e. those who migrated to Australia since 2008. Semi-structured qualitative interviews were conducted in Australia in July–August 2018. In an era of increased emigration, Australia has become a key destination for Irish trained doctors [9, 40]. Ethical approval for the study was obtained from the institutional research ethics committee. All interviews were conducted by the lead author (29 face to face, 11 by telephone). A snowball sampling strategy was utilised, whereby potential respondents made contact in response to an article published in the emigrant section of an Irish national newspaper in 2018 [46]. Respondents were Irish doctors who had trained and/or worked in Ireland and were now working in Australia (see Table 1 for details). Interviews lasted an average of 49 min (ranging from 28 to 95 min) and interviews continued until data saturation was reached. The interview schedule was organised around five major themes: qualifying and working as a doctor in Ireland; the decision to migrate; working and living in Australia; and future plans. Interviews were digitally recorded and transcribed by a third party.

**Table 1:** Irish trained doctors in Australia respondent table (*N* = 40)

Gender	Male	27
	Female	13
Year of arrival	2016–2018	15
	2008–2015	25
Age on arrival	20–29	22
	30–35	12
	Over 35	6
Career stage at migration	After internship	18
	During postgraduate medical training	20
	Consultant	2
Location in Australia (at time of interview)	Melbourne/Victoria	17
	Perth/Western Australia	14
	Adelaide/South Australia	4
	Other location Australia	5
Migration status (at time of interview)	Temporary working visa	15
	Permanent residency	16
	Citizenship	6
	Unknown	3
Future intentions	Intend to return to Ireland	14
	Intend to remain in Australia	16
	Undecided	10
Career stage/grade (at time of interview)	Resident Medical Officer (RMO)	10
	Trainee	16
	Consultant	14
Current specialty	Pre-Speciality/Resident Medical Officer (RMO)	12
	Emergency Medicine	7
	Surgery	5
	Anaesthesia	4
	Other specialty	12

Data analysis was conducted using MaxQDA software. Analysis of the transcripts began with a process of open coding whereby broad codes were attached to important sections of the transcript text [47]. The largest examples of these broad codes were ‘work environment Ireland’ and ‘work environment Australia’. The process of developing more analytical and theoretical codes [47, 48] initially involved mapping respondent experiences in both Ireland and Australia to the five levels of the Health Professional Wellness hierarchy developed by Shapiro et al. [49]. Discussions with co-authors at this point led to a refined focus on job quality, the Irish health system and the triggers for migration (‘migrant type’ code). Analytical codes were developed, with reference to the literature on job quality and extreme work (e.g. [20, 22]) and on employee responses to a deterioration in job quality, i.e. exit, voice [16]. This helped to frame the doctors’ use of emigration to Australia as exit [16] from an austerity-constrained Irish health system and as a means of recovering high job quality.

Although the focus of this paper is on their experiences in the Irish health system, it is important to note that, as International Medical Graduates (IMGs) in Australia, Irish trained doctors face practice restrictions for their first 10 years of working. These restrictions encourage IMGs to work in ‘areas of need’ which restricts their access to the private sector. Once they have served in an ‘area of need’ for 5 years, they are entitled to apply for permanent residency [50]. For this reason, the public/private distinction is less than it might otherwise be.

## Results

Only 14/40 respondents intended to return to work in the Irish health system, which indicates a break with the traditional return migration pathways of an earlier generation of doctors and which echoes the findings of a 2014 survey which found that only one in four emigrant doctors surveyed intend to return to work in Ireland [51]. Respondents’ descriptions of their work in Irish hospitals were aligned with characteristics of extreme jobs [20, 22] and include (1) long hours and fast pace; (2) inordinate scope of responsibility; doing more with less and 24/7 availability; and (3) always fighting a climate of negativity. Their decisions to exit [16] from the Irish health system are also explored in relation to (4) emigration as exit and (5) exit and voice.

1. Long hours and fast pace

Long working hours and fast pace were frequently mentioned by respondents as negative aspects of working as a hospital doctor in the Irish health system.

*‘My very first week as an intern, I did 80 hours. . . spread across 5 days’* (Respondent 28).

*‘that environment . . . everyone is very stressed and ... It's just not a nice environment. Just people aren't happy in it. You're doing long hours, people are sicker because they're not being looked after as well as they should be, but that's not the on-the-ground people's fault, it just the system’* (Respondent 19).

Respondents spoke about the challenge of working long hours in fast-paced work environments within which it was difficult to adequately care for themselves, even in terms of taking adequate rest breaks. They described an extreme work context where everyone was ‘flat-out working really hard’ (Respondent 28). They also spoke about the risks that this way of working posed to their physical and mental health and safety.

*‘after working for 29 hours . . . straight . . . I got maybe an hour or two sleep, and then my boss asked me to operate with him . . . I went to operate with him, but then I drove home and crashed my car’* (Respondent 32).

2. Inordinate scope of responsibility, doing more with less and 24/7 availability

Beyond long hours, respondents described excessive workloads which they attributed to understaffing. One respondent likened their workplace to ‘a war zone’ (Respondent 49). Others explained the challenge of trying to keep up with the workload:

*‘I felt like I spent my whole day putting out fires. I didn't ever get on top of the workload. The system didn't work . . . I felt continuously stressed. I felt that I never got on top of the patient case load. My consultants were there but they were very remote’* (Respondent 5).

The feeling of being understaffed and overworked was prevalent throughout interviews, with respondents reporting that it negatively impacted on the care they could provide their patients; ‘I can't do the best for my patients because I just don't have. . . the space’ (Respondent 14).

*‘After hours, which is the scariest bit . . . you're pretty much left on your own. I remember running around the hospital with ECG's, trying to find a senior doctor to just, “Would you mind looking at this? I think this person's really sick.” . . . when you're doing 24-hour call, and you're just running around for 24 hours putting out fires and just keeping them alive until Monday. It would scare you* (Respondent 23).

The combination of intense working, long working hours and 24/7 availability extended into their personal lives in other problematic forms. Respondents frequently reported that they felt they could not take time off work, even when they were sick, as there was no one to cover their workload in their absence.

*‘You'd come to work dying before you call in sick, because you don't want to let your colleagues down And if you did call in sick, everyone's like, “Oh, God. You're sick.” And then you come back the next day and they're like, “You weren't that sick. You shouldn't have called in sick.”’* (Respondent 21).

Other respondents were inadequately supported by their employer/hospital to take bereavement or paternity leave, as these respondents explain;

*‘When my Mum passed away, I had asked to take a week off work . . . I got how many emails from admin? . . . a memo of what the entitlements were for the death of a family member. . . I did try to come into work and I got sent home and I still got these emails saying I'm not entitled to that’* (Respondent 48).

*‘My wife had complications and I was rostered to be on in the obstetric hospital. . . Rather than get someone else to cover my shift, they’ll roster me in the intensive care unit, so if she deteriorated it would mean [that it was] my problem anyway’* (Respondent 1).

For these two respondents, feeling unsupported by their employer during major life events strongly influenced their decision to exit the Irish health system.

3. Always fighting a climate of negativity

Respondents described how the combination of long working hours and busy work environments resulted in work-related stress and anxiety:

*‘I was . . . quite unhappy and probably a hard person to live with because I was under immense stress’* (Respondent 11).

*‘It’s probably just part and parcel of being an intern, but I met friends of mine crying in side rooms off of wards just in bits about what just happened’* (Respondent 8).

Disrespectful behaviour from colleagues, both medical and non-medical, had an impact on the work environment and created a negative atmosphere at work. Respondents who had experienced or had witnessed such behaviour, had accepted it as the norm at the time.

*‘the bigger the hospital, the angrier people were. A lot of aggression, a lot of fighting, just because everyone is exhausted and burnt out’* (Respondent 49).

*‘I don't think we need sympathy, we just need a fair deal, that's all. . . the way it's lined up everyone's overworked, that's the hospitals in general: the nurses are overworked, the doctors are overworked. It means that there's a lot of antagonism all the time’* (Respondent 43).

A related issue was that respondents felt that they did not have adequate time to care for colleagues who were struggling; ‘everyone's just busy trying to just keep their own mental health up, so they're not great at looking after others’ (Respondent 13). This meant that those in need of support, of collegiality, were not always able to access it: ‘nothing terrible . . . ever happened, but I just felt secluded and alone’ (Respondent 18).

A further source of negativity was the challenge of negotiating overtime payments. This was an area of conflict between hospital administrators and junior hospital doctors. As hospitals were eager to ensure compliance with the European Working Time Directive^2^, junior hospital doctors were routinely underpaid for the hours they had worked:

*‘When I was an intern, I worked hundred-hour weeks as a standard . . . the administrator . . . would pay maybe 83 of the hours that week’* (Respondent 13).

*‘The hospital I worked in, I did 24-hour shifts. In fact, it was even worse than that. You'd only get paid for a 24-hour shift, but you'd have to do 26 hours. And you'd just be not paid for two hours because they couldn't be seen to break the 24-hour rule’* (Respondent 23).

4. Emigration as exit

Deteriorating job quality and the normalisation of extreme working influenced the decision to emigrate. This coincided with (and was reinforced by) austerity-related staffing and funding cutbacks within their hospital work environments. These impacted on senior, or consultant hospital doctors, as well as on junior colleagues or trainees, as this respondent explains:

*‘the deteriorating quality of the job . . . we were having to take responsibility for . . . juniors that weren’t up to scratch and for doing extra work for colleagues’* (Respondent 31).

Respondents felt that working as a hospital doctor in Ireland put them at risk of burnout and that they needed to exit the Irish health system, or the medical profession, to find more sustainable ways of working.

*‘I was going to leave medicine before I came out [to Australia]. . . I was burnt out. I was definitely suffering burnout when I moved out here. Yeah, no question, after my year as a Reg, I was definitely suffering burnout after that year. And it was a job I loved doing. . . it was with a team that I loved working with, but just gets too much’* (Respondent 49).

*‘The system. Yeah, I left Ireland to recharge, get out of there. I was really dissatisfied with how my career was going. I didn't see myself being three years into my career after working so hard in medical school to do so well and publish to many papers, and then to kind of turn around and be like, what's gone wrong? . . . Yeah, it was just to try and . . . Self-preservation basically’* (Respondent 22).

5. Exit and voice

All respondents were emigrant doctors who, by definition, had already exited the Irish health system at the time of interview. Most respondents (38/40) held junior hospital doctor posts within the Irish health system immediately prior to their emigration. Some explained that they had opted for exit rather than voice because of their precarious employment circumstances.

*‘in every hospital we're temporary staff. . . So, all that needs to happen is the contract doesn't need to be renewed’* (Respondent 49).

As junior hospital doctors, known in the Irish context as NCHDs (non-consultant hospital doctors), respondents were relatively junior members of the medical profession who held short-term employment contracts within the hospital. Perhaps as a result of this, voice was considered an isolating activity which would carry significant career risks:

*‘In order for us to stay and rectify some of these issues would require someone taking the hospitals to task on it, but that requires someone standing up . . . you put your head above that stand, it’s chopped off and that’s it. Your career is done . . . you can’t complain too loudly, or it will definitely negatively impact your career’* (Respondent 49).

*‘This . . . clash with one of the professors . . . No one really standing up for what they privately thought was really disgraceful, but obviously, I guess, the culture is there . . . you don’t say anything’* (Respondent 13).

Another deterrent to voice is that it can fail to influence change. Several respondents had voiced dissatisfaction with the Irish health system prior to exit, citing their involvement in grassroots or union campaigns for change, or simply by highlighting the reasons for their exit.

*‘I left Ireland as noisily as I could. I did two radio interviews on the day I was leaving . . . I was in the newspaper, blah, blah’* (Respondent 17).

There was a sense of inevitability from respondents when the anticipated change failed to materialise and the decision to emigrate was taken. One respondent felt that their exit from the Irish health system might, paradoxically, influence health system change:

*‘one of my reasons for leaving was I might do a better service by leaving, help drag it down, and the quicker we drag it down, the quicker we can build it back up again’* (Respondent 38).

In this way, they felt that exit could contribute to the health system collapse they felt was a prerequisite for change.

## Discussion

### Extreme ways of working

Respondents described hospital workplaces in Ireland that were understaffed and overstretched and within which stressful and adversarial work relationships had become normalised. Based on Buchanan et al.’s criteria [22], our findings suggest that respondents’ descriptions of working as hospital doctors in Ireland align with the characteristics of extreme working. Interestingly, ‘making life or death decisions’ [22] while an everyday feature of hospital medicine, was not widely discussed by respondents in determining their decision to leave the Irish health system. Instead tipping points related to working hours and pace, breaks, leave and treatment at work: organisational factors which had altered the boundaries of work in unsustainable ways. Findings are suggestive of hospital workplaces within which extreme has become the new normal.

*‘Extreme becomes normal in mundane, prestigious or “mainstream” workplaces as more organizations become overstretched. “Extreme” is common and socially acceptable, and professionals are . . . expected to find a way to cope with or even embrace this new normal’* [21].

The deterioration in job quality and working conditions occurred against a backdrop of historic underfunding [15], combined with post-2008 austerity which decreased the health budget. Importantly, we reiterate that rather than remain in the Irish health system and cope with extreme working, or accept it as the new normal, our respondents opted to emigrate.

### Austerity and doctor emigration

The challenge posed by doctor emigration for the Irish health system is twofold—firstly that the traditional pattern of short-term doctor migration for postgraduate training associated with a high likelihood of return, appears to have been altered in the decade since 2008. The reasons for emigration have changed, with dissatisfaction with the Irish health system [8] and with the quality of jobs available in that system now driving emigration. The likelihood of return is also changing, with only 1 in 3 respondent doctors (*N* = 40) intending to return to work in the Irish health system, echoing the findings of a 2014 survey which found that only 1 in 4 emigrant Irish trained doctors (*N* = 307) intended to return [51]. Secondly doctor emigration reduces staffing levels in the Irish health system, thereby increasing the workloads of those who remain. As previous studies have shown [52, 53], this can make them more susceptible to extreme working and, perhaps, emigration. It is difficult—but imperative—to see how this ‘vicious circle’ [52] of emigration, understaffing and extreme working might be broken. In this regard, the experiences in Ireland mirror those in other EU countries impacted by austerity, including Romania [12] which has seen working conditions, low salaries and discontent with the health system, act as ‘push’ factors driving doctor emigration, and Portugal [13], which has seen salary reductions and public sector downsizing since 2009. The availability of better working conditions and salaries internationally [3, 13] are also driving doctor emigration flows. The similarities across countries highlight the extent to which doctor emigration flows may be altered by unanticipated ‘shocks’ to the system, such as the 2008 economic recession and austerity.

### Exit and voice

Doctor emigration is an individual solution to the unsolved health system problem of deteriorating job quality and the normalisation of extreme work. Migrating to Australia enabled Irish doctors to escape extreme working and improve their job quality, while continuing to work in hospital medicine. This illustrates that ‘exit’ is a viable option to Irish trained doctors, as highly skilled, English-speaking migrants whose skills are in demand globally.

Emigration as the preferred solution to deteriorating job quality could be considered as a failure of voice, defined as ‘any attempt at all to change, rather than to escape from, an objectionable state of affairs’ [16]. The high costs associated with voice—linked to career risks—deterred junior hospital doctors from using it [28] and cast exit (via emigration) as a more reliable means of improving their job quality. Hirschman describes this as a ‘victory of . . . mobility over politics’ [26], i.e. doctors migrate because they do not believe that they will successfully influence change in the health system and/or because their attempts at voice have not succeeded and they cease to believe that improvement is likely [28]. If they have little hope of improvement (in the health system, in their job quality), then they will be less likely to take the risk of using their voice to attempt to bring about change. In an era of reduced union activity, opportunities for collective voice have lessened and ‘responsibility for creating better jobs has shifted . . . onto the shoulders of individuals’ [54], a situation likely reinforced by the individualised nature of medicine and medical careers (Byrne JP, Conway E, McDermott A, Matthews A, Prihodova L, Costello R, Humphries N: Same Medicine, Different Conditions: A qualitative exploration of the psychosocial structures of medical work in Ireland and Australia, in preparation).

In relation to voice, a distinction could be drawn between junior and senior hospital doctors (consultants), as they have different status within the health system which likely impacts on their propensity to use voice and perhaps their likelihood of success. Although both junior and senior hospital doctors experience the strain of extreme working and deteriorating job quality in the Irish health system since 2008, the authors anticipate that their differing status within the hospital and wider health system may influence their capacity to inform health system change. In 2013, junior hospital doctors in Ireland used their collective voice to launch a high-profile campaign to cap their working hours. The campaigns, ‘enough is enough’ and ’24 no more’ [55]—successfully generated publicity and support and culminated in a 1-day strike by junior hospital doctors in 2013 [55]. Although improved, concerns about long working hours and ‘nominal rather than actual compliance with the ETWD requirements’ [56] continue to be voiced by hospital doctors to this day. Although junior hospital doctors successfully employed their collective voice in that instance, the health system failure to resolve the issue of long working hours may have strengthened the belief that that voice is unlikely to yield change, thereby further encouraging junior hospital doctors to opt for exit/emigration rather than (the riskier option of) voice.

Hirschman warns that people tend to ‘underestimate the effectiveness of voice when exit is dominant’ [16]. This has significant implications for the Irish health system. Firstly, because the success of the exit option (emigration) may deter voice and encourage further emigration; Secondly, because hospital doctors may become resigned to extreme working and accept it as the new normal. Thirdly, the dominance of exit over voice poses a challenge to healthcare organisations which *rely* on employee voice to provide ‘organisational intelligence about patient safety hazards and . . . quality of patient care’ [57]. If hospital doctors have little faith that their voice will inform improvements in the health system, either in relation to job quality, working conditions, or patient safety, will they use voice? The need to better support junior hospital doctors ‘to understand and speak up about safety concerns’ [58] was highlighted by a recent study into reporting patient safety concerns in Ireland.

In this regard, it will be interesting to see the extent to which two 2019 campaigns for change in the Irish health system, will succeed. The campaigns focussed on pay equality and patient safety (#carecantwait and #fightforfairness) and are led by senior hospital doctors, with the backing of the Irish Hospital Consultants Association (IHCA) and the Irish Medical Organisation (IMO). Although the campaigns give voice to similar issues, i.e. patient safety and improved pay and conditions for hospital doctors, the involvement of senior hospital doctors and the backing of their representative bodies may enhance the likelihood that voice successfully informs change.

## Conclusion

Respondent doctors are exiting the Irish health system because of a deterioration in medical job quality to the point of extremity. Health systems and employers must mitigate the emergence of extreme work in healthcare to retain doctors and ensure that health systems can continue to provide healthcare. But what can be done in response? In relation to medical job quality, Shapiro et al. recommend that it can be strengthened (and wellbeing promoted) by attending to job quality at five levels, beginning with the most basic—ensuring access to rest breaks, providing doctors with a safe work environment and access to job security, showing appreciation and adequately compensating doctors and enabling them to contribute to medicine to the best of their ability [49]. The themes raised by our respondents could also be used to inform workplace interventions to create more supportive work environments for hospital doctors, which enable them ‘to deliver excellent care for their patients while, at a minimum, not paying an overwhelming personal price’ [49].

In relation to doctor emigration, Hirschman described the point in Irish history at which Irish mass emigration came to be seen as a threat to the State rather than a ‘safety valve’ [27] and how, in response, 1950’s Ireland sought ‘to meet the challenge of mass exit by changing the underlying conditions that had resulted in the outflow’ [27]. This logic could also be applied to doctor emigration today, which poses a significant threat to the Irish health system. The question is whether there is a willingness to change the underlying conditions that are driving this exodus of doctors from Ireland.

## Data Availability

The datasets generated and/or analysed during the current study are not publicly available due to privacy/confidentiality concerns. Reasonable requests for access can be made to the corresponding author who will consider any such requests in collaboration with the RCPI research ethics committee.

## Footnotes

1 Later papers from the HDRM project will focus on wellbeing and hospital doctors and the drivers of migration for Irish hospital doctors in Australia.

2 The European Working Time Directive was introduced in 2004 and intended to protect workers, ensuring that they did not work in excess of 48 h per week.
